# Randomized controlled trial of Family Nurture Intervention in the NICU: assessments of length of stay, feasibility and safety

**DOI:** 10.1186/1471-2431-13-148

**Published:** 2013-09-24

**Authors:** Martha G Welch, Myron A Hofer, Raymond I Stark, Howard F Andrews, Judy Austin, Sara B Glickstein, Robert J Ludwig, Michael M Myers

**Affiliations:** 1 Department of Psychiatry, Columbia University College of Physicians & Surgeons, New York, NY, USA; 2 Department of Pediatrics, Columbia University College of Physicians & Surgeons, New York, NY, USA; 3 Department of Developmental Neuroscience, New York State Psychiatric Institute, 1051 Riverside Drive, Unit 40, New York, NY, 10032, USA; 4 Mailman School of Public Health, Columbia University, New York, NY, USA; 5 EB Sciences, Oakland, CA, USA

**Keywords:** Premature infant, NICU, Nurture, Safety, Feasibility, Length of stay

## Abstract

**Background:**

While survival rates for preterm infants have increased, the risk for adverse long-term neurodevelopmental and behavioral outcomes remains very high. In response to the need for novel, evidence-based interventions that prevent such outcomes, we have assessed Family Nurture Intervention (FNI), a novel dual mother-infant intervention implemented while the infant is in the Neonatal Intensive Care Unit (NICU). Here, we report the first trial results, including the primary outcome measure, length of stay in the NICU and, the feasibility and safety of its implementation in a high acuity level IV NICU.

**Methods:**

The FNI trial is a single center, parallel-group, randomized controlled trial at Morgan Stanley Children’s Hospital for mothers and their singleton or twin infants of 26–34 weeks gestation. Families were randomized to standard care (SC) or (FNI). FNI was implemented by nurture specialists trained to facilitate affective communication between mother and infant during specified calming interactions. These interactions included scent cloth exchange, sustained touch, vocal soothing and eye contact, wrapped or skin-to-skin holding, plus family-based support interactions.

**Results:**

A total of 826 infants born between 26 and 34 weeks during the 3.5 year study period were admitted to the NICU. After infant and mother screening plus exclusion due to circumstances that prevented the family from participating, 373 infants were eligible for the study. Of these, we were unable to schedule a consent meeting with 56, and consent was withheld by 165. Consent was obtained for 150 infants from 115 families. The infants were block randomized to groups of N = 78, FNI and N = 72, SC. Sixteen (9.6%) of the randomized infants did not complete the study to home discharge, 7% of those randomized to SC and 12% of FNI infants. Mothers in the intervention group engaged in 3 to 4 facilitated one- to two-hour sessions/week. Intent to treat analyses revealed no significant difference between groups in medical complications. The mean length of stay was not significantly affected by the intervention.

**Conclusion:**

There was no significant effect demonstrated with this intervention amount on the primary short-term outcome, length of stay. FNI can be safely and feasibly implemented within a level IV NICU.

**Trial registration:**

Clinicaltrials.gov: NCT01439269

## Background

Survival rates of preterm infants have increased with improved care and technology over the past 20 years. Yet, the risks for long-term adverse neurodevelopmental and behavioral outcomes remain unacceptably high. These include attention deficits [1], executive dysfunction [2,3], depression and psychotic disorders [4], and autism spectrum disorder [5]. Accordingly, there have been increasing calls for novel evidence-based interventions that can that can limit or overcome long-term developmental morbidities that accompany preterm birth [6,7], as well as for more rigorous randomized controlled trials (RCTs) to validate the results [8]. In addition to several new pharmaceutical and medical interventions, there have been many interventions aimed at improving outcomes for the infant through enrichment of the infant’s NICU environment, including increased parent involvement in infant care [9]. The best studied of these latter approaches are Newborn Developmental Care and Assessment Program (NIDCAP) [10,11], skin-to-skin care [12,13], and massage therapy [14-16]. However, for a variety of reasons that may include cost of implementation, demand on resources, insufficient evidence for long-term effectiveness and resistance to change, these NICU interventions have yet to be universally adopted.

The Family Nurture Intervention (FNI) protocol was designed to induce a connection between the mother and her preterm infant as early as possible and to engage support from the husband and family members in these efforts in order to alter the developmental trajectory of the preterm infant. Our hypothesis was that one of the earliest consequences of this change in trajectory would be a decrease in length of stay.

While some aspects of FNI are similar to other mother-infant interventions, several aspects make FNI novel. FNI focuses on the mother and infant as a dyad and therefore seeks to positively effect a change in the co-regulatory relationship between infant and mother. FNI does this by facilitating affective communication and an emotional connection between the two. It does so in the very early stages of NICU care when infants are confined to the incubator by using scent cloth exchange, sustained touch, vocal soothing and eye contact. At later stages, when the infants are stable and can remain outside the incubator, FNI facilitates wrapped or skin-to-skin holding and as much engagement of mothers in daily infant care as possible. Throughout the hospitalization, FNI facilitates family-based support for mother-infant interactions.

There are several notable differences between our intervention and trial design and those mentioned above. None of these studies have examined the effects of facilitated affective communication between mother and infant using an RCT design. Furthermore, no other trial has specifically identified the “Calming Cycle” [17] as the central intervention feature. And, in surveying the literature, few trials incorporate strategies that increase mother-infant interactions early in the NICU while the infant is confined to the isolette, fewer still that aim at providing the mother and family with a parenting strategy that can be employed throughout infant development at home.

In this first report of our RCT, we present the primary outcome (length of stay; LOS) [17,18]. Length of stay was chosen because we thought it would be an early maker for effectiveness of the intervention, and because it has been an important outcome variable for other NICU intervention studies, including NIDCAP, kangaroo care and massage therapy [8,13,16], though results were not always consistent. In addition, the cost/day of moderately preterm infants (32–34 weeks GA) has been estimated at $1734/day [19]. Thus, any decrease in LOS associated with FNI could present a significant economic benefit. In contrast, an increase in LOS could negatively impact adoption of the intervention.

This first report also addresses concerns raised prior to the start of the trial about the safety of FNI for this fragile patient group and the possibility that this intervention could increase infection and complications due to increased touch and scent cloth exchange. Thus, assessing safety and feasibility was an essential component of this trial and results will guide the development of NICU interventions by us and others, and at the same time expand our understanding of what minimal risk approaches can be tolerated by premature infants, their parents and, very importantly, the NICU staff.

We report here that FNI did not significantly affect length of stay in the NICU. Results show that FNI is practicable within a level IV NICU environment and is safe for preterm infants born between 26 and 34 weeks of gestation.

## Methods

This study is a single center, parallel-group RCT. Full details of the trial design, methods, outcome measures, and hypotheses have been published elsewhere [17]. A sample size of 150 (75 per group) was projected to have power of 1-*β* = .8, with significance level *α* = .05 and a medium effect size of approximately d = 0.4. This was determined to be sufficient to detect a group difference of 5.3 days in length of hospital stay from admission to the NICU until discharge home [18], and also gave sufficient power for secondary outcome analyses [17].

As reported in the manuscript, all infants included in this RCT were between 26 and 35 GA. The original protocol restricted the gestational ages of the infants to 26 to 32 weeks. After a few months, we decided to increase the age to 35 weeks in order to increase enrollment. At the same, we decided to conduct a small pilot study in late preterm infants (34 - 35 weeks) using the same intervention and protocol. We therefore modified the recruitment protocol to include infants between ages of 26 and 34 weeks. There were no deviations in implementation of the original or amended approved protocol.

### Subjects

Mothers gave informed consent for their infants’ and their own participation.

Preterm infants born at Morgan Stanley Children’s Hospital at Columbia University Medical Center and admitted to the NICU were eligible for the study if they were 1) born between 26 and 35 weeks postmenstrual age, 2) free of major congenital defects, 3) singleton or twin gestation and, 4) above the third percentile of weight for gestational age at birth. Infants were excluded if their mother 1) had a history of drug dependence, psychosis or other severe mental health problems, 2) could not speak adequate English for obtaining consent, 3) was younger than 18 years of age, 4) did not have support from at least one additional adult in the home, 5) was too ill to participate. Additional factors that prevented participation were: 1) anticipated infant discharge within less than 10 days, 2) inability of the mother to meet the visitation goal of 4 times/week, 3) inability of the family to participate in post-discharge follow-up, 4) prior enrollment of the mother and/or infant in a competing study, 5) withholding of approval by attending physician, 6) infant death.

### Randomization

The study coordinator performed randomization and group assignment. Block randomization between Intervention (FNI) and Standard Care (SC) groups was employed to maintain equivalence of group sizes prospectively. Prepared and numbered envelopes (six assignments per block) were selected consecutively for group assignment, with a single draw for twins. Twins were assigned jointly to either intervention or control groups because it was deemed neither ethical nor feasible to expect a mother to adopt different nurturing approaches for each of her twins. No stratification by gestational age or twin status was used in the randomization procedure.

### Blinding

Blinding of intervention team, nursing and medical staff was not possible in this trial; Nurture Specialists had to know to whom they were to administer the intervention, and the study staff had to know from which mothers certain data were to be collected. Blinding of the mothers was also not possible, given that all mothers were in the same NICU and knew in advance their group assignment.

### Intervention

FNI is based on the hypothesis that adverse consequences of prolonged separation of mother and infant following preterm birth can be ameliorated by a dyadic intervention comprising repeated experience with calming activities. These activities occur during calming sessions that are facilitated by Nurture Specialists, former NICU nurses trained in implementing the FNI protocol. Nurture Specialists also involve and engage family members in reassuring and calming the mother and in providing continued support for her when the infant was discharged.

Calming session activities engage the mother and infant reciprocally in physical, sensory and emotional experiences. Each session was comprised of as many of the calming procedures as possible or warranted by the infant’s distress. Specific methods used in FNI are described briefly below and, in more detail, in a prior publication [17]. Prior to engaging in any of the FNI activities, mothers and family members were instructed in infection prevention procedures. The FNI calming procedures were as follows.

#### Scent cloth exchange

Mothers were instructed to exchange special 12 by 16 inch cotton cloths with their infants on a daily basis. The mother’s scent cloth was worn close to her skin overnight and was placed with the infant each day; the infant’s scent cloth was placed in bed with the infant during the preceding 24 hours and was taken home each day by the mother.

#### Calming touch

Mothers were shown how to calm their infants using firm sustained touch by cupping one hand around the infants’ legs and feet and placing the other hand on the abdomen. While engaging in calming touch, the Nurture Specialist prompted the mother to communicate her thoughts and emotions to the infant in her native language. During all of these activities the mother was encouraged to seek and maintain eye contact with her infant as much as possible.

#### Holding

When infants could be taken out of the incubator, the Nurture Specialists assisted the mother to engage in skin-to-skin or wrapped calming sessions. Nurture Specialists aimed to facilitate holding sessions at least four times per week; however, mothers were encouraged to engage in all calming techniques at all of their visits.

Although feeding is a critical mother-infant interaction, feeding was not included as one of the facilitated mother-infant activities because the NICU employed a feeding specialist for all mothers. However, the nurture specialists encouraged mothers to feed, change, and bathe their infants. Fathers were also encouraged to engage in these activities.

### Standard care

Mothers in the Standard Care (SC) group followed hospital protocol. SC mothers were able to engage in nurturing activities of their choosing, which in this NICU included skin-to-skin or non-skin-to-skin holding. These activities of SC mothers were optional and not documented by the study staff; however, SC mothers recorded these activities on a self-report visit log.

### Outcome measures

#### Intervention fidelity

The amount of the intervention varied according to the needs and availability of the infant, the mother and the family. Mothers recruited into the study and assigned to the FNI group were asked to complete four facilitated one-hour intervention calming sessions per week for the duration of the NICU stay. In addition, mothers were asked to exchange scent cloths with their babies upon each NICU visit for the duration of the NICU stay. However, FNI mothers who consulted with our nurture specialists more frequently performed more FNI. We targeted a minimum weekly amount of FNI knowing that target duration (weeks) was dependent on infants’ LOS. Implementation of the mother-infant activities during each visit was documented on check lists by the Nurture Specialists. Mother-infant activities in the NICU, with or without Nurture Specialist support, were also self-recorded by FNI mothers with the aid of a NICU visit log. Given the limitations of self-report data, potentially compounded by changes to the data collection format during the trial, outcomes reported here are limited to scent cloth exchanges per week (FNI group only) and frequency and duration of holding sessions, either in or out of the isolette.

#### Safety

Safety of the intervention was determined by examining specific clinical characteristics of infants during hospitalization and at discharge (Table 1). These parameters including infant mortality, infections and gastrointestinal, neurological and cardiac disorders, were obtained by study staff from hospital records.

**Table 1:** Clinical characteristics of infants during hospitalization and at discharge: N = 134 infants who completed the study

	**SC**	**FNI**		
	**N = 67**	**N = 67**		
	**n (%)**	**n (%)**	***χ***^**2**^	***p***
During NICU stay				
Antibiotics to rule out sepsis	40 (59.7)	43 (64.2)	.285	.594
Treated for presumed sepsis	7 (10.4)	9 (13.4)	.284	.594
Confirmed sepsis	11 (16.4)	7 (10.4)	1.027	.311
Medical treatment for NEC	4 (6.0)	7 (10.4)	.891	.345
Surgical treatment for NEC	2 (3.0)	0 (0.0)	*	.496
Caffeine	9 (13.4)	7 (10.4)	.284	.594
Intra-ventricular hemorrhage	17 (25.4)	14 (20.9)	.378	.539
Hydrocephalus	2 (3.0)	0 (0.0)	*	.496
Seizures diagnosed	1 (1.5)	0 (0.0)	*	1.00
Treatment for seizures	1 (1.5)	0 (0.0)	*	1.00
Cardiology †	7 (10.4)	7 (10.4)	0.000	1.00
Retinopathy of prematurity: diagnosis	6 (9.0)	7 (10.4)	.085	.770
Retinopathy of prematurity: surgery	1 (1.5)	0 (0.0)	*	1.00
Feeding problems	15 (22.4)	7 (10.4)	3.481	.062
At discharge				
Nasal oxygen	2 (3.0)	0 (0)	*	.496
Other medications	16 (23.9)	12 (17.9)	.722	.395
	**mean ± SD**	**mean ± SD**	***t***	***p***
Weight (grams)	2596 (748)	2521 (565)	.655	.513

*Fisher’s exact test.

† 4 Atrial septal defects; 2 ventricular septal defects; 5 patent foramen ovale; 1 ventricular dilatation; 1 dysplastic pulmonary valve; 1 biventricular dilatation.

*Abbreviations:NEC* Necrotizing enterocolitis.

#### Length of stay

The primary outcome of the RCT, Length of Stay (LOS), was the duration of hospitalization from date of birth to date of discharge home [18]. Measurement of LOS for infants discharged elsewhere (i.e., not home) ceased on the day of discharge. These cases are included in the intent-to-treat analysis.

### Statistical methods

Data from the infant, family and nurture assessment measures were entered via Citrix application server software into a central customized Scientific Information Retrieval (SIR) database and exported into SPSS v20 for analysis. In the intent-to-treat analysis, LOS was compared for the two study groups using survival analysis: the Kaplan-Meier estimator and the log-rank test. Data collection ceased prior to discharge home for infants who were transferred to another hospital, died or withdrew from study (data right censored). FNI subject participation was assessed by frequency and duration of Nurture Specialist-supported calming sessions. Comparisons between groups with regard to calming activities were made with Student’s t-tests. With regard to missing data, we took a conservative approach. In the case that data were missing it was assumed there was no activity or event. These cases were assigned a null value for the analysis.

### IRB

Ethical approval for this study was obtained from the Institutional Review Board (IRB) of Columbia University Medical Center and was in accordance with the Helsinki Declaration. An independent Data and Safety Monitoring Board (DSMB) was appointed to oversee the implementation of the study. The trial is registered at ClinicalTrials.gov with registration number NCT01439269.

## Results

### Recruitment and retention of subjects

**Figure 1:**
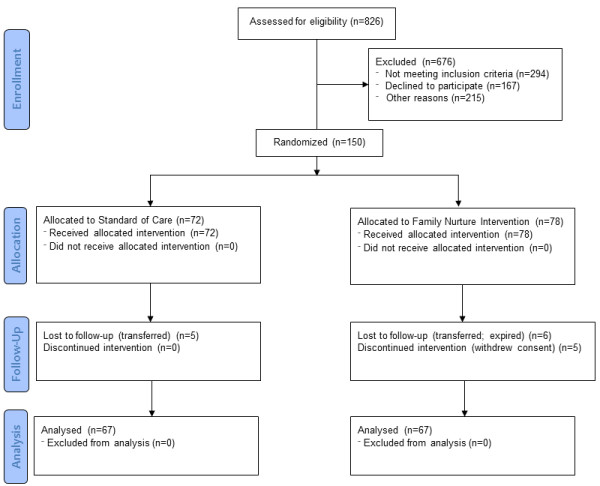
Study consort chart.

Enrollment for the FNI-NICU study began in January 2009. Recruitment ended in June of 2012 and the last infant in the study was discharged in September of 2012. The recruitment cascade for the study is given in Figure 1. There were 826 age-eligible infants admitted to the NICU during the study period. Of these, 124 infants were excluded for infant-related exclusion criteria (congenital anomalies and <3rd percentile for weight). An additional 170 infants were excluded for the following maternal indications: non-English speaking (97), postpartum illness (21), psychiatric history (15), substance abuse history (10), age <18 years (10), and no adult support at home (17). Of the remaining 532 infants, no consent was attempted for 159 infants due to an inability to participate. These participation exclusions included: discharge was anticipated in 10 days or less (117), family was moving out of the area and would not be able to participate in follow-up (20), attending physician did not approve approach to consent (18), infant died (3), infant was enrolled in another study that precluded second enrollment (1). Of the remaining 373 infants, consent was not obtainable because the mother was not available to schedule a consent meeting (56). Of the remaining eligible infants consent was denied for 167: not interested (34), overwhelmed (29), father refused (30), did not want to commit the required time (29), never agreed after repeated contact (45). Consent was obtained for 150 infants from 115 families. The infants of these families were randomized to the two trial arms (72 Standard Care and 78 FNI infants). The unequal distribution of infants was due to a greater number of families with twins being randomized to the FNI arm of the trial.

Overall, N = 16 (10.6%) infants were lost to follow-up (LTFU), prior to achieving the primary endpoint (5 SC and 11 FNI). This left 67 infants in each group who were discharged home. In the SC group, all 5 LTFU infants were discharged to another hospital. In the FNI group, 1 infant died, 5 were discharged to another hospital and, 5 infants had consent withdrawn; 2 (twins) due to the fathers’ concerns about infections, 1 because the father did not want the infant enrolled in a research study, and 2 twins because the mother was not able to meet the time/visit commitments required.

### Demographics and clinical information

Baseline demographic information for study participants is presented in Table 2. Clinical information is provided in Table 3. There were no significant differences between groups. Consistent with risk of pre-term delivery, antenatal treatment with steroids was high (93%), as was use of labor suppressants (82%). Consistent with the study design, the mean gestational age of enrolled infants was between 30 and 31 weeks for both groups. Overall, the rate of C-section for singleton gestations was not different for SC and FNI mothers (55% vs. 59%). However, C-sections for twin gestations were less frequent in the SC group (81% vs. 100%). The rate of C-section in singletons is slightly higher than the national average in preterms as of 2006 [20], which at the time was trending upward.

**Table 2:** Demographic characteristics of 115 families randomized for study

**Family characteristics**	**SC**	**FNI**
	**N = 56**	**N = 59**
	**Mean ± SD**	**Mean ± SD**
Mothers’ age (years)	32.8 (5.69)	34.1 (6.11)
Fathers’ age (years)	34.9 (6.49)	37.3 (8.09)
	**n (%)**	**n (%)**
Married	39 (69.6)	41 (69.5)
Mothers’ ethnicity		
Black	13 (23.2)	13 (22.0)
Hispanic	14 (25.0)	17 (28.8)
White	24 (42.9)	24 (40.7)
Other	5 (8.9)	5 (8.5)
Fathers’ ethnicity		
Black	15 (26.8)	12 (20.3)
Hispanic	12 (21.4)	15 (25.4)
White	21 (37.5)	28 (47.5)
Other	8 (14.3)	4 (6.8)
Mothers’ education		
High school or lower	6 (10.7)	7 (11.9)
Some college	15 (26.8)	12 (20.3)
Graduate or higher	35 (62.5)	40 (67.8)
Fathers’ education		
High school or lower	14 (25.0)	13 (22.0)
Some college	11 (19.6)	12 (20.3)
Graduate or higher	31 (55.4)	34 (57.6)
Family income		
< $40,000	13 (23.2)	10 (16.9)
$40,000 - $70,000	3 (5.4)	13 (22.1)
> $70,000	34 (60.7)	31 (52.5)
Did not report	6 (10.7)	5 (8.5)

**Table 3:** Baseline clinical characteristics: N = 115 mothers of N = 150 infants

**Baseline characteristics**		**SC**	**FNI**
		**N = 56**	**N = 59**
**Maternal**		**n (%)**	**n (%)**
Preeclampsia		17 (30.4)	23 (39.0)
HELLP syndrome		3 (5.4)	4 (6.8)
Hypertension		10 (17.9)	9 (15.3)
Diabetes		9 (16.1)	13 (22.0)
Steroids		52 (92.9)	55 (93.2)
Tocolytic drugs		47 (83.9)	47 (79.7)
Cesarean delivery		35 (62.5)	43 (72.9)
		**SC**	**FNI**
		**N = 72**	**N = 78**
**Infant**		**mean ± SD**	**mean ± SD**
Gestational age (wk)		30.7 (2.6)	30.8 (2.1)
Birth weight (g)		1474 (439)	1426 (396)
Length at birth (cm)		39.6 (4.2)	39.7 (3.4)
Head circumference at birth (cm)		28.1 (3.0)	28.2 (3.0)
		**n (%)**	**n (%)**
Male		36 (50.0)	41 (52.6)
Singleton		40 (55.6)	39 (50.0)
Cesarean delivery		48 (66.7)	62 (79.5)
Resuscitated at birth		18 (26.5)	21 (29.2)
Placed on CPAP at delivery		67 (94.4)	67 (90.5)
Apgar scores	≥7 at 1 minute	57 (79.2)	54 (69.2)
	≥4 at 1 minute	66 (91.7)	69 (88.5)
	≥7 at 5 minutes	68 (94.4)	72 (92.3)
	≥4 at 5 minutes	71 (98.6)	74 (94.9)

*Abbreviations:CPAP* Continuous positive airway pressure, *HELLP* syndrome Hemolysis, elevated liver enzymes, low platelet count.

### Intervention fidelity

Intervention activity was reported in two ways. Nurture Specialists recorded each session with the mother. In addition, mothers recorded sessions with their infants in a log. According to the Nurture Specialist records, facilitated calming sessions occurred on average 3.5 times per week (median = 3.7; IQR = 2.7 – 4.1) over the course of the infants’ hospitalization. These sessions were divided amongst scent cloth exchanges (median = 2.6 times per week, IQR = 1.9 – 3.7), sessions of vocal soothing and comfort touch (median = 3.2 times per week, IQR = 2.4 – 3.8), and skin-to-skin holding (median = 1.9 times per week, IQR = 1.3 – 2.7). The overall number of facilitated calming sessions was slightly below the planned minimum of 4 times per week. However, 84% of sessions were >1 h in duration (average 1.6 h/session or 6.4 h/week), which slightly exceeded our target of 4 h/week of facilitated FNI activities. Based on an average LOS of 47.4 days (see LOS section), the average total duration of FNI facilitated activities was 43 hours per subject. Weekly rate of visits did not differ by mother’s educational group (F = .049, *p* = .953). The weekly rate of visits also did not differ by infant’s gestational age (F = −.242, p = .09).

According to the Mother’s logs, there was little, if any, difference in the amount of specified nurture activities from those facilitated by the Nurture Specialist. Mothers’ logs reflected more frequent skin-to-skin holding sessions by FNI mothers (FNI 2.0 ± 0.17; SC 0.8 ± 0.14 times per week, p < 0.001) on average. When these activities did take place, there were of longer duration (FNI 83 ± 5; SC 63 ± 4 minutes/session, p < 0.01) on average. Mothers in the FNI group reported scent cloth exchanges at an average rate of 2.7 ± 0.2 times per week. FNI mothers also reported more frequent infant diaper changes on average than SC mothers (FNI 3.9 ± 0.46; SC 2.5 ± 0.23 times per week; p = 0.01).

### Safety

The incidence of infant mortality during the study was 1 death in each study group. The death in the FNI group occurred in the Columbia NICU. The baby was a twin who developed a volvulus with bowel necrosis progressing to overwhelming sepsis and expired within 24 hours of surgical bowel resection. The other twin had no infectious complications and was discharged home. The death in the SC group occurred in an outside NICU to which the baby had been transferred when the family relocated. The cause of death was attributed to unresolved chronic lung disease while in a NICU to which the infant had been transferred.

The incidence of major pathological diagnoses recorded for the 134 infants discharged from the Columbia NICU are summarized in Table 1. There were no significant differences between the FNI and SC groups in the diagnosis or treatment for sepsis, necrotizing enterocolitis, neurological conditions, renal disorders or retinopathy of prematurity. There was a non-significant trend for increased diagnosis of patent ductus arteriosis (PDA) in FNI infants (p = .055) but not for surgical closure of the ductus or other cardiac conditions. There was a trend toward a decreased incidence of feeding problems in the FNI group (p = .062). There was evidence at discharge of severe bronchopulmonary dysplasia in 2 SC infants (discharged on nasal cannula oxygen). No FNI infants were discharged on oxygen.

### Length of stay (LOS)

All 150 infants were included in an initial intent-to-treat analysis; 16 infants who were not discharged to their home (transferred to other NICUs) were considered as LTFU and their data were right-censored at the date of last contact, or death [21]. All other infants (N = 134) were followed until discharge home from our NICU. LOS ranged from 5 to 212 days, with a median of 35.5 (IQR 20 – 60) days. Kaplan-Meier survival analysis (time to event, discharge home) produced an estimated mean (±SD) LOS of 47.4 ± 27.4 days for the FNI group and 50.8 ± 45.8 days for the SC group. Although mean LOS was 3.4 days shorter in the FNI group, median LOS was 4 days longer in the FNI group (SC median = 36, 95% CI = 29 – 43; FNI median = 40, 95% CI = 30 – 50) with no significant difference between the groups (Mantel-Cox log-rank *χ*^*2*^ = 0.163, df = 1, *p* = .687). Controlling for gestational age at birth, a proportional hazards model revealed no significant association between LOS and amount of FNI (expressed as a weekly rate of facilitated visits) in intervention group infants (*β* = .959, Wald *χ*^*2*^ = .340, df = 1, *p* = .560).

## Discussion

The overall goal of our NICU research program is to determine whether perinatal and long-term development can be improved by the earliest possible implementation of biologically important mother-infant interactions in the NICU. While we found no impact of FNI upon LOS, the primary outcome of this trial, we did find that the intervention was safe and feasible to implement within a high-acuity, level IV NICU environment. In brief, we found that mothers were willing to engage in FNI activities and that these activities could be done within the context of a level IV NICU. In addition, we found the intervention did not increase medical risks. Although FNI encouraged skin-to-skin holding, which has been shown to decrease LOS in preterm infants [13], the intervention also includes interactions not previously well studied in preterm infants (e.g. scent cloth exchange, and repeated novel calming interactions, and family sessions). Therefore, we were attuned to the possibility that FNI might either decrease or, perhaps increase, LOS. Contrary to both alternatives, results from the study showed that infants randomized to FNI showed no significant difference in LOS, compared with infants receiving standard care.

In order to support a broader implementation of FNI in other NICUs, it was important to determine that the intervention would not interfere with standard care, that it could be carried out fully and that it was medically safe. Some doctors and nurses expressed concerns that infants in the intervention group would be subjected to increased risk of infection and potentially other adverse medical consequence associated with repeated removal of the infants from the controlled environment of the incubator or bassinet. Clinical outcomes during the course of stay in the NICU and at the time of discharge proved these concerns to be unwarranted. There was no evidence for any increase in sepsis, necrotizing enterocolitis, seizures, retinopathy, or in the need for oxygen or greater medication in the FNI group.

We found that intervention mothers met with the nurture specialists on average 3.5 times a week for slightly greater than one hour per session and that they willingly engaged in all of the FNI calming activities. The high rate of study retention in the FNI group after consent (86%) suggests that the intensive involvement required of participating mothers did not deter participation. FNI mothers did engage in skin-to-skin holding for greater duration and frequency and, as a further measure of maternal involvement we quantified the number of times the mother changed her baby’s diapers, which was significantly increased in the FNI group.

We found that LOS was not significantly affected by FNI. In our study, mothers in the FNI group engaged in skin-to-skin care, on average, less than 1 hour/day. This is considerably less than reported for previous studies of intermittent skin-to-skin care in which significant effects of LOS were found. In these studies mothers engaged in skin-to-skin care for between 1 and 6 hours each day [22-24]. Another important difference between these prior studies and ours was the range of birth weights of the infants enrolled. In one of these, all babies enrolled were less than 1500 g [24], and in the other two, the standard deviation of birth weights in the study infants was between 110 and 120 g [22,23], whereas in our study the SDs of birth weights were nearly 4 times greater (SC = 439 g; FNI = 396 g). Had infants been exposed to a higher dose of maternal scent via the scent cloth, or if mothers had engaged in more frequent and longer skin-to-skin sessions and other FNI calming activities, we might have obtained a significant effect on LOS. There was a large variance in LOS in both groups (SC standard deviation = 46 days, FNI standard deviation = 27 days) largely due to a wide range of gestational ages. Thus, the effect on LOS might have been reduced had we enrolled infants with a narrower age range.

### Limitations

A significant limitation of this trial was that a large number of families declined to participate. This was largely due to the considerable study burden imposed by the trial: for both groups, the protocol included multiple time consuming physiological and neurobehavioral assessments during the NICU stay and during multiple follow-up visits over a two-year period; for the intervention group, mothers were additionally asked to meet regularly with the Nurture Specialists throughout the NICU stay and the families were asked to meet once prior to discharge. In future studies of FNI, we plan to reduce study burden by separating the intervention portion of the study from the follow-up. We believe this will significantly help to reduce the refusal rate. There was no indication that anyone refused because they did not want to do nurture. Therefore, we do not see the low percentage of study enrollment itself as an impediment to intervention acceptance in the NICU.

Another limitation to consider is that the sample might be biased towards those who had better support systems, more time and fewer competing responsibilities. We believe this to be a limitation that worked against us. These mothers would be more similar to each other than to the rest of the population and more likely to give lots of attention to the baby, whichever group they were assigned to. So, the self-selected sample would make it harder to show an effect, not easier.

Contamination is a potential limitation in this study. It was impossible to prevent some communication between subjects in the FNI and SC groups. However, the great majority of the mothers on our 80 bed capacity unit were not in the study. Usually, there were fewer than 3 study mothers in the NICU, thus limiting the chances of contamination; the maximum number of enrolled subjects at one time was 4 FNI mothers and 2 SC mothers. Occasionally, SC mothers expressed regret at their group designation. One SC mother tried scent cloth exchange with her infant.

A limitation of this report is that longer-term follow-up assessments of neurophysiologic and cognitive and behavioral development were not available. However, they are on-going and will continue until at least two years of age. These longer-term assessments will be used to test the hypothesis that FNI leads to improved neurobehavioral outcomes of prematurely born infants. Nevertheless, we have documented improved EEG activity in FNI infants compared with SC infants at near to term [25], which supports that FNI may indeed be associated with improved neurodevelopmental outcomes.

## Conclusions

FNI did not significantly affect the primary outcome of this study, LOS in the NICU. Nonetheless, this trial did demonstrate that FNI is safe in a high acuity NICU. In addition, we found that mothers were willing to engage in the study intervention activities, and that FNI was compatible with routine NICU activities.

## Abbreviations

FNI: Family nurture intervention; LOS: Length of stay; LTFU: Lost to follow-up; NICU: Neonatal intensive care unit; RCT: Randomized controlled trial; SC: Standard care.

## Competing interests

The authors declare that they have no competing interests.

## Authors’ contributions

MGW and MMM conceived of the study, shared in the responsibility for the conduct of the RCT, and participated in the preparation of all drafts of the manuscript. MGW designed the interventions and trained the personnel who facilitated the interventions. MAH advised on the trial design and methodology and participated in the preparation of the final drafts of the manuscript. RIS advised on the methodology, acted as liaison with medical staff and participated in the preparation of the manuscript. HA advised on data management and participated in the preparation of the manuscript. JA designed and performed statistical and power analyses and participated in the preparation of the manuscript. RJL advised on the trial design and methodology and participated in the preparation of the final drafts of the manuscript. MMM advised on methodology and data acquisition and participated in the writing all drafts of the manuscript. The FNI Trial Group advised on recruitment, data acquisition, data analysis, methodology and/or preparation and editing of the manuscript. All authors read and approved the final manuscript.

## Authors’ information

MGW is Assistant Professor of Clinical Psychiatry (Division of Developmental Neuroscience), and Pathology & Cell Biology and Pediatrics. She is a board certified child psychiatrist who has treated children with a wide range of developmental and behavioral disorders for more than 35 years with family nurture therapy.

MAH is a Professor Emeritus and founder of the Division of Developmental Psychobiology (now Developmental Neuroscience) in the Department of Psychiatry at Columbia University and New York State Psychiatric Institute. He was the first Director of the Sackler Institute for Developmental Psychobiology at Columbia University.

RIS is Professor of Pediatrics and Obstetrics and Gynecology. He is the past Director and Principal Investigator for the Perinatal Emphasis Research Center from the National Institute of Child Health & Development. In his collaboration with the NICU Study, Dr. Stark engaged in the study design and methods. Dr. Stark brings years of clinical and translational experience to the study team, providing insights on patient care, sleep studies, and EEGs.

HFA is an Associate Clinical Professor of Neuroscience and Biostatistics at the Mailman School of Public Health, Columbia University, and Director of the Data Coordinating Center at New York State Psychiatric Institute and Columbia University Medical Center. Dr. Andrews has extensive experience in the design of data systems and statistical analysis for clinic trials.

JA is a registered psychologist, a doctoral candidate in the Epidemiology Department, Mailman School of Public Health, Columbia University, and data analyst with the FNI-NICU study. Ms. Austin has a broad experience of research, monitoring and evaluation of large international programs supporting reproductive and maternal health.

SBG is currently the Director of Elegant Brain Sciences. Dr. Glickstein has conducted many studies of brain development and behavior in animal models. Dr. Glickstein participates in literature research and manuscript preparation and editing.

RJL is Managing Director of the BrainGut Initiative and supervises staff involved in the Initiative’s studies and research. Mr. Ludwig also actively participates in manuscript preparation and editing.

MMM is Chief of the Division of Developmental Neuroscience in the Department of Psychiatry at New York State Psychiatric Institute. He has conducted numerous studies in both animal models and human infant related to development of physiological systems and behavior. He is past president of the International Society of Developmental Psychobiology.

FNI Trial Group: Ladan Afifi, Amy Bechar, Beatrice S. Beebe, Susan A. Brunelli, Kathryn E. Carnazza, Christine Y. Chang, Patricia A. Farrell, Ewelina S. Fiedor, Qais Karim, Shanna Kofman, Yasmine A. Koukaz, Mary T. McKiernan, William P. Fifer and Sorana Sopterian are members of the Department of Psychiatry, Columbia University College of Physicians & Surgeons, New York, NY, USA. David A. Bateman, Phillip G. Grieve, John M. Lorenz, Richard A. Polin and Rakesh Sahni are members of the Department of Pediatrics, Columbia University College of Physicians & Surgeons, New York, NY, USA. David P. Merle is a member of the Mailman School of Public Health, Columbia University, New York, NY, USA. Amie A. Hane is a member of the Department of Psychology, Williams College, Williamstown, MA, USA.

## Pre-publication history

The pre-publication history for this paper can be accessed here:

http://www.biomedcentral.com/1471-2431/13/148/prepub
